# Treatment outcome and associated factors among patients with epilepsy

**DOI:** 10.1038/s41598-018-35906-2

**Published:** 2018-11-26

**Authors:** Yirga Legesse Niriayo, Abraham Mamo, Tesfaye Dessale Kassa, Solomon Weldegebreal Asgedom, Tesfay Mahari Atey, Kidu Gidey, Gebre Teklemariam Demoz, Seid Ibrahim

**Affiliations:** 1 Department of Clinical Pharmacy, School of Pharmacy, College of Health Sciences, Mekelle University, Mekelle, Tigray Ethiopia; 2 Clinical Pharmacy and Pharmacy Practice Unit, Departments of Pharmacy, College of Health Sciences, Axum University, Axum, Tigray Ethiopia

## Abstract

Epilepsy is a major public health problem worldwide. Despite multiple drug therapies, people with epilepsy continue to have frequent seizures. There is a dearth of data on epilepsy treatment outcome and associated factors in our setting. Therefore, the aim of this was to assess treatment outcome and associated factors among epileptic patients on follow up at the neurologic clinic of Ayder comprehensive specialized hospital, Ethiopia. A cross-sectional study was conducted on randomly selected epileptic patients. Data were collected through patient interview and review of medical records. Epilepsy treatment outcome was evaluated in terms of seizure control status in the last one year follow up period. Binary logistic regression analysis was performed to identify predictors of treatment outcome. A total of 270 patients were included. Of whom, 46.6% had controlled seizures. Whereas, 38.5%, 8.8%, and 5.9% had experienced seizure attacks 1–5 times, 6–10 times, and greater than 10 times, respectively. Alcohol consumption [adjusted odds ratio [(AOR): 14.87, 95% confidence interval (CI): 3.25–68.11], negative medication belief [AOR: 3.0, 95%CI: 1.31–6.71], low medication adherence [AOR:11.52, 95%CI: 3.25–40.82], and presence of comorbidities [AOR: 10.35, 95%CI: 4.40–24.40] were predictors of uncontrolled seizure. Our finding revealed that more than half of the epileptic patients had uncontrolled seizure. Epileptic patients with a negative medication belief, comorbidities, low medication adherence, and those who consume alcohol were more likely to have uncontrolled seizure. Therefore, more emphasis should be given to these patients.

## Introduction

Epilepsy is a chronic neurologic disorder characterized by repeated epileptic seizures attacks which result from paroxysmal uncontrolled discharges of neurons within the central nervous system^1–4^. The definition of epilepsy requires the occurrence of at least one epileptic seizure^3^. Epilepsy is a major public health problem that affects more than 50 million people worldwide, of whom, 80% were living in developing countries^5–8^. African countries are among the highly affected regions and it is estimated that ten million people live with epilepsy in Africa^7,8^. Likewise, Ethiopia is affected by epilepsy with a reported prevalence of 5.2/1000 population and annual incidence of 64 per 100,000 population^9,10^.

Antiepileptic Drugs (AEDs) can be indicated for Patients who have had one or more epileptic seizures. The choice of therapy for the management of epilepsy varies depending on the type, frequency, and severity of the seizures^4,11^. Making an accurate diagnosis of the type of epilepsy is crucial to select the best therapy^8,11^. Majority of epileptic seizures are controlled with the optimal use of the currently available AEDs. However, about one-third remained uncontrolled despite optimal therapy^12,13^. Although most of the people with epilepsy can become seizure-free with the optimal use of drug therapy, the treatment outcome in the majority of epileptic patients remains unsatisfactory in resources limited countries^8^. Studies have shown that majority [80–90%] of the patients with epilepsy are not receiving appropriate treatment in developing countries^8,14^.

A number of problems affect the provision of adequate treatment of epilepsy and these problems are more pronounced in developing countries. The major problems include; lack of qualified medical personnel, unavailability of medications, poor community knowledge and awareness, cultural beliefs, stigma, poor economy, lack of prioritization, and poor health system infrastructure^14–16^. Many studies have shown that inappropriate drug therapy and non-adherence were the leading causes of poor seizure control^17–19^.

Several factors have been found to be associated with treatment outcome in epilepsy. These include; gender, age of seizure onset, type of epilepsy, seizure frequency, etiology of epilepsy, duration of epilepsy, electroencephalography abnormality and presence of comorbidities^14,20–22^. Poorly controlled seizure leads to impairment of quality of life, excessive bodily injury, neuropsychological impairment, social stigma, reduced marriage rates, poor education, reduced employment levels, and finally shortened lifespan^23–25^.

Assessment of epileptic patient’s treatment outcome and its predictors is crucial to develop treatment optimization strategies and responsible care of patients as clinicians may have difficulty in identifying patients that are less likely to have controlled seizure. Although different studies have been conducted in different parts of the world, there is no adequate data on epilepsy treatment outcome and associated factors in Ethiopia. To our knowledge, there is no study in our particular setting. Hence, our study investigated the treatment outcome and associated factors among epileptic patients.

## Methods

### Study design, study setting and study period

A hospital-based cross-sectional study was conducted from March 2017 to May 2017 at the neurologic clinic of Ayder comprehensive specialized hospital (ACSH), which is the second largest public hospital in Ethiopia with a catchment population of about 10 million people. The study period was from March 2016 to May 2017.

### Study participants

Adult patients (age ≥ 18 years) with the diagnosis of epilepsy who have been on regular follow- up for at least one year with at least one AED were included in the study. Patients were recruited into the study during their appointment for medication refilling. Patients were excluded if they had a follow-up period of less than one year, seriously ill to complete the interview, refused to give consent, and those with incomplete medical records. A total of 270 patients were included in the study using simple random sampling technique.

### Data collection instrument and procedure

All consented epileptic patients who visited the hospital during the data collection period and fulfilled the inclusion criteria were included in the study. Data regarding sociodemographic, medication adherence, medication belief and experience were retrieved by interviewing patients using the standardized questionnaire. Respective medical and medication records were retrieved by reviewing patient’s medical record chart using data abstraction checklist. The clinical information of the patients during the last one year follow up period (starting from the date of interview during the data collection period until the last one year) were assessed. All patient were followed for one year to determine their clinical and treatment related characteristics We trained the data collectors about the objective of the study, methods of data collection including data extraction from patient charts as well as techniques of interviewing patients.

Patients’ belief about their medication was assessed using the belief about medicines questionnaire (BMQ), which has been validated for use in deferent chronic illness group studies^26,27^. It is a self-reported questionnaire that contains two five-item scales assessing patients’ belief about the necessity of the prescribed medications for controlling their illness and their concerns about the potential adverse consequences of taking it. Accordingly, participants were considered to have strong medication necessity belief if the average sum of the five-item medication necessity scale score (ranging from 5–25) is above the midpoint. Conversely, if the score is below this point they were considered to have low medication necessity belief. Similarly, participants were considered to have strong concern belief about their medication adverse effect if the average sum of the five-item medication concern scale score (ranges from 5–25) is above the midpoint, otherwise, they were considered to have low medication concern belief. The overall patients’ belief about their medication is obtained by subtracting the average 5–item medication concerns scale score from the average sum of 5–item patient’s medication necessity scale score. If the difference is positive, the patient is said to have positive medication belief. Conversely, if it is negative, the patient is said to have negative medication belief.

Medication adherence was assessed using Morisky’s medication adherence scale, which has been validated for use in chronic illness adherence assessment^28^. It is a self-reported questionnaire which contains eight adherence related questions, in which the total score ranges from 0 to 8 points. The degree of adherence was determined according to the score resulting from the sum of all items. Accordingly, medication adherence was considered as low, medium, and high if the total score is <6, 6 to <8, and 8 points, respectively.

Epileptic patients were defined and identified according to the international league against Epilepsy (ILAE)^3^. Accordingly, the definition of epilepsy requires the occurrence of at least one epileptic seizure. Participants who had any chronic disease other than epilepsy were considered to have comorbidity. Epileptic patients were said to have psychiatric disorder comorbidity if they had confirmed diagnosis of psychiatric disorders such as depression, schizophrenia, mood disorders, and anxiety by psychiatrist according to Diagnostic and Statistical Manual of Mental Disorders (DSM-5)^29^.

Treatment outcome was measured in terms of seizure control status and seizure frequency. In order to evaluate epilepsy treatment outcome, seizure status of the patients in the last one-year follow-up period was considered. Every patient was followed for one year to determine the frequency of seizure in the one-year follow-up period. Operationally, the seizure status was considered to be controlled if the patient had not experienced any seizure attacks in the last one year, and not controlled if the patient experienced one or more seizure attacks in the last one year follow up period.

### Data analysis

Data were recorded into an EPI data management (version 4.2.0) and analyzed using the Statistical Package for the Social Science (SPSS version 21.0). Descriptive analysis was computed using frequency and mean (standard deviation, SD) for categorical and continuous variables, respectively. The frequency of seizure control status was determined. Multicollinearity was checked to test correlation among predictor variables using variance inflation factor (VIF) and none was collinear. A VIF < 8 was considered as a cut point for excluding collinearity. Independent variables with p < 0.2 in univariable binary logistic regression analysis were re-entered into a multivariable binary logistic regression model to identify predictors of treatment outcome in epilepsy. A p value of < 0.05 was considered statistically significant in all analyses.

### Ethical approval and informed consent

This study was approved by the institutional review board (IRB) of Mekelle University, College of Health Sciences. The aim and protocol of the study were fully explained to all patients included in the study and written informed consent was obtained from all participants. The privacy of individual information was strictly preserved. All the methods were performed in accordance with approved institutional guidelines.

## Results

### Sociodemographic characteristics of the study participants

A total of 270 epileptic patients were included in this study and analyzed. Of whom, 62% were males and the mean (±SD) age was 30.31 ± 10.95 years. Majorities of the participants were unemployed (73%) and urban dwellers (60%). A large proportion of the participants attended primary and secondary school (30.7% and 51.9%, respectively). With regard to social drug use, 6.3%, 4.1%, and 4.4% of the participants were using alcohol, khat, and cigarette, respectively (Table 1).

**Table 1:** Socio–demographic characteristics of the participants (n = 270).

Characteristics	Number (%)
Gender, male	168(62)
Age in years	
18–30	163(60.4)
31–60	99(36.6)
>60	8 (3)
Residence, urban	162(60)
Educational level	
Illiterate	29 (10.7)
Primary education	83(30.7)
Secondary education	140(51.9)
College and above	18(6.7)
Marital status	
Married	70(25.9)
Single	162(60)
Divorced	24(8.9)
Widowed	14(5.2)
Employment status	
Employed	73(27)
Unemployed	197(73)
Social drug use	
Abstain	230(85.2)
Alcohol	17(6.3)
khat	11(4.1)
Cigarette	12(4.4)
Monthly income (in Ethiopian Birr)	
<=1500 birr	141(52.2)
>=1500 birr	129(47.8)
Age at the time of first seizure	
<=15	77(28.5)
16–30	108(40)
31–45	61(22.6)
46–60	21(7.8)
>60	3(1.1)

### Medication belief and adherence status of the participants

Our study reported that majority (70%) of the participants had strong necessity belief towards the importance of their medication while 35% had strong concern belief. Overall, 70% had a positive belief about their medication. More than half (51.5%) of the patients had low adherence to their prescribed medications (Table 2).

**Table 2:** Medication belief and adherence status of the participants (n = 270)

Characteristics	Number (%)
Medication necessity belief	
Strong necessity belief	189(70)
Low necessity belief	81(30)
Medication concern belief	
Strong concern belief	94(35)
Low concern belief	176(65)
Overall medication belief	
Negative belief	81(30)
positive belief	189(70)
Level of medication adherence	
High adherence	27(10)
Medium adherence	104(38.5)
Low adherence	139(51.5)

### Disease and treatment related characteristics

The mean (±SD) duration of epilepsy was 5.42 ± 3.08 years and the median (IQR) was 5(3–7) years with a range from 1 to 20 years. More than half (59.6%) of the participants had lived with epilepsy for five or more years and 37.8% had one or more comorbidities. The most commonly identified comorbidity among epileptic patients was psychiatric disorder (20.4%). Generalized tonic-clonic seizure (GTCS), 84.4% was the most commonly diagnosed type of epilepsy. Nearly half (48.5%) of the study participants were on monotherapy of AEDs. Our finding reported that 43% of the patients complained about adverse drug events (ADEs) related to their medication (Table 3).

**Table 3:** Clinical and treatment related characteristics of the participants (n = 270).

Characteristics	Number (%)
Presence of comorbidity	
No	102(37.8)
Yes	168(62.2)
Commonly identified co morbidities	
Psychiatric disorder	55(20.37)
Migraine headache	20(7.4)
Hypertension	15(6)
Human immune deficiency virus (HIV)	11(4)
Others	16(6)
Duration of epilepsy in years	
Mean ± SD	5.42 ± 3.08
Median (IQR)	5(3–7)
<5	109(40.4)
≥5	161(59.6)
Type of seizure	
GTCS	220(81.5)
Focal seizure	36(13.3)
Absence seizure	5(1.9)
Unclassified seizure	9(3.3)
Number of AED(s)	
One	131(48.5)
Two	125(46.3)
Three	14(5.2)
ADE	
Yes	117(43.3)
No	153(56.7)

GTCS, General tonic-clonic seizure, AED, Anti-epileptic drug, ADE, Adverse drug event, SD, standard deviation, IQR, interquartile range.

### Seizure frequency and treatment outcome

Out of the total, 46.6% participants had controlled seizure. Whereas, 38.5%, 8.8%, 5.9% had experienced seizure attacks 1–5 times, 6–10 times, and greater than 10 times, respectively (Table 4).

**Table 4:** Distribution of seizure frequency and seizure status of the participants (n = 270).

Frequency of seizure during the last one year	Number (%)
**0**	126(46.6)
1–5 times	104(38.5)
6–10 times	24(8.8)
>10 times	16(5.9)

### Factors associated with treatment outcome

Using univariable binary logistic regression analysis, epileptic patients with controlled seizure and uncontrolled were compared using the socio-demographic, disease and medication related characteristics. Accordingly, alcohol consumption [Crude odds ratio (COR): 4.51, 95% confidence interval (CI): 1.26–16.11], negative medication belief [COR: 3.28, 95%CI: 1.86–5.79], low medication adherence [COR: 16.69, 95%CI: 5.82–47.87], presence of comorbidities [COR: 3.54, 95%CI: 1.47–8.44], triple AED therapy [COR: 3.54, 95%CI: 1.47–8.44], and ADE [COR: 3.58, 95%CI: 2.16–5.95] were significantly associated with uncontrolled seizure (Table 5).

**Table 5:** Univariable logistic regression analysis of factors associated with treatment outcome of epileptic patients (n = 270).

Variables	Treatment outcome		COR (95% CI)	p-value
	Controlled seizure, n (%)	Uncontrolled seizure, n (%)		
Gender, female	42(15.5)	60(22.2)	1.43(0.87–2.35)	0.160
Age category				
18–30	70(26)	93(34)	1	1
31–60	51(19)	48(18)	0.71(0.43–1.17)	0.178
>60	5(2)	3(1)	0.45(0.10–1.95)	0.287
Age at seizure onset				
<=15	33(12.2)	44(16.3)	1	1
16–30	50(18.5)	58(21.5)	0.87(0.48–1.57)	0.643
31–45	30(11.1)	31(11.5)	0.78(0.40–1.52)	0.459
>45	13(4.8)	11(4.1)	0.64(0.25–1.59)	0.333
Residence, urban	75(27.8)	87(32.2)	1.04(0.637–1.69)	0.881
Marital status				
Married	70(26)	92(34)	1	1
Single	37(13.7)	33(12.2)	1.47(0.84–2.59)	0.177
Divorce	10(3.7)	14(5.2)	1.57(0.62–4.01)	0.346
Widowed	9(3.3)	5(2)	0.62(0.19–2.05)	0.435
Education				
Illiterate	13(4.8)	16(5.9)	1.54(0.47–5.02)	0.475
Primary	38(14.1)	45(16.7)	1.48(0.53–4.13)	0.453
Secondary	65(24.1)	75(27.8)	1.44(0.54–3.87)	0.467
Tertiary	10(3.7%)	8(3)	1	1
Employment status				
Unemployed	92(34.1)	105(38.9)	0.99(0.58–1.70)	0.985
Employed	34(12.6)	39(14.4)	1	1
Income (in Ethiopian birr)				
=<1500	62(23.0)	79(29.3)	1.26(0.78–2.03)	0.354
>1500	64(23.7)	65(24.1)	1	1
Duration of epilepsy				
<5 year	48(17.8)	61(22.6)	1.19(0.73–1.95)	0.476
≥5 year	78(28.9)	83(30.7)	1	1
Alcohol use				
No	123(45.6)	130(48.1)	1	1
Yes	3(1)	14(5.2)	4.51(1.26–16.11)	0.020
Smoking				
No	120(44.4)	139(51.5)	1	1
Yes	6(2.2)	5(2)	0.81(0.24–2.71)	0.726
Khat chewing				
No	122(45.2)	136(50.4)	1	1
Yes	4(1.5)	8(3)	1.93(0.57–6.59)	0.293
Medication belief				
Positive belief	104(38.5)	85(31.5)	1	1
Negative belief	22(8.2)	59(21.9)	3.28(1.86–5.79)	<0.001
Medication adherence				
High adherence	22(8.2)	5(2)	1	1
Medium adherence	75(27.8)	29(10.7)	1.70(0.59–4.92)	0.326
Low adherence	29(10.7)	110(40.7)	16.69(5.82–47.87)	<0.001
Co–morbidity				
No	115(42.6)	53(19.6)	1	1
Yes	11(4.1)	91(33.7)	17.950(8.87–36.34)	<0.001
Type of seizure				
GTCS	101(37.4)	119(44.1)	1	1
Focal seizure	18(6.7)	18(6.7)	0.85(0.42–1.72)	0.648
Absence seizure	2(0.7)	3(1)	1.27(0.21–7.76	0.794
Unclassified seizure	5(1.9)	4(1.5)	0.68(0.18–2.60)	0.572
Number of AEDs				
One	68(25.2)	60(22.2)	1	1
Two	50(18.5)	59(21.9)	1.34(0.80–2.23)	0.266
Three	8(3)	25(9.3)	3.54(1.47–8.44)	0.004
ADE				
No	73(27)	40(14.8)	1	1
Yes	53(19.6)	104(38.5)	3.58(2.16–5.95)	<0.001

COD, Crude odds ratio, CI, Confidence interval, AED, antiepileptic drugs, ADE, adverse drug event, GTCS, Generalized tonic-clonic seizure.

On further multivariable binary logistic regression model; Alcohol consumption [adjusted odd ratio (AOR): 14.87, 95% CI: 3.25–68.11], negative medication belief [AOR: 3.0, 95%CI: 1.31–6.71], low medication adherence [AOR: 11.52, 95%CI:3.25–40.82], and presence of comorbidities [AOR: 10.346, 95%CI: 4.387–24.399] were found to be predictors of uncontrolled seizure (Table 6).

**Table 6:** Multivariable logistic regression analysis of factors associated with treatment outcome among epileptic patients (n = 270).

Predictors	Treatment out come		AOR (95%CI)	p-value
	Controlled seizure, n (%)	Uncontrolled seizure, n (%)		
Gender, female	42(16)	60(21.9)	1.78(0.87–3.71)	0.114
Age category				
18–30	70(26)	93(34)	1	1
31–60	51(19)	48(18)	0.79(0.22–2.92)	0.726
>60	5(2)	3(1)	1.79(0.05–62.2)	0.749
Marital status				
Married	70(26)	92(34)	1	1
Single	37(13.7)	33(12.2)	1.55(0.40–6.08)	0.526
Divorce	10(3.7)	14(5.2)	1.24(0.31–5.01)	0.761
Widowed	9(3.3)	5(2)	0.73(0.05–9.74)	0.808
Alcohol use				
No	123(45.6)	130(48.1)	1	1
Yes	3(1)	14(5.2)	14.87(3.25–68.1)	<0.001
Medication belief				
Positive belief	104(38.5	85(31.5)	1	1
Negative belief	22(8.2)	59(21.9)	3.00(1.301–6.71)	0.009
Medication adherence				
High adherence	22(8.2)	5(2)	1	1
Medium adherence	75(27.8)	29(10.7)	2.46(0.70–8.77)	0.166
Low adherence	29(10.7)	110(40.7)	11.52(3.25–40.82)	<0.001
Comorbidity				
No	115(42.6)	53(19.6)	1	1
Yes	11(4.1)	91(33.7)	10.35(4.40–24.40)	<0.001
Number of AEDs				
One	68(25.2)	60(22.2)	1	1
Two	50(18.5)	59(21.9)	1.09(0.51–2.32)	0.819
Three	8(3)	25(9.3)	1.84(0.57–5.96)	0.310
ADE				
No	73(27)	40(14.8)	1	1
Yes	53(19.6)	104(38.5)	2.132(0.891–5.102)	0.089

AOD, Adjusted odds ratio, CI, Confidence interval, AED, antiepileptic drugs, ADE, adverse drug event.

## Discussion

Currently, therapeutic advances have resulted in meaningful changes in the diagnosis and management of epilepsy^30^. However, the practice of epilepsy management is inconsistent in different countries depending on the available expertise and resource^8^. Although evidence has shown that a greater proportion of epileptic patients become seizure free with the optimal use of the available AEDs^8,11–13^, less than half of the patients remain seizure free in our study. This could be attributed to the lack of qualified medical personnel, unavailability of medications, poor community knowledge and awareness, and poor health system infrastructure in our setting where resources are limited. Our finding is also quite different from a study done in Gonder, Ethiopia^31^ in which 82% of the epileptic patients achieve seizure remission over 3 months follow-up period. This variation could be due to the difference in follow-up period (3 months vs. 12 months). In line with our study, the majority of the patients had uncontrolled seizure in other similar studies^32–34^.

Several studies revealed that alcohol consumption is a risk factor for developing seizure and it increased the risk of seizure in epileptic patients^35–38^. Similarly, alcohol was found to be a predictor of uncontrolled seizure in our study. This could be explained that alcohol consumption could lead to sleep deprivation, missing meals, missing medications and increase the side effect of AED which were reported as triggering factors of seizure^14,35–38^. Hence, much should be done on awareness of alcohol use of epileptic patients.

Our finding reported that low medication adherence was significantly associated with uncontrolled seizure. In agreement to our study, many studies have shown that non-adherence to medication was the leading cause poor epilepsy control^14,17,19,31,32,39^. In addition, our study revealed that patients with a negative medication belief were less likely to have seizure free period compared to those with a positive medication belief. The possible justification for this could be patients with a negative medication belief are less likely to adhere to their medications as revealed by our study and other similar studies^40,41^. Hence, educational programs should be designed to improve the perception of patients about their medication as well as their medication adherence. Our study also reported that epileptic patients with comorbidity were less likely to have controlled seizure than those without comorbidities which is in line other similar studies^41–43^. Thus, more emphasis should be given to these patients.

Patients with a triple AED therapy and those who experienced ADEs were less likely to have seizure-free period though statistically not significant on the multivariable regression model. Even though evidence-based guidelines recommend the use of monotherapy for the majority of epileptic patients^41,44,45^, only 48.5% were maintained on monotherapy in our study. Overall, utilization of monotherapy was found to be low compared to other similar studies^14,46,47^. This could be due to the absence of specific epilepsy treatment guideline and lack of expertise of healthcare professionals in our setup. Our study reported that 43% of the patients had experienced ADE related to their AED therapy. This finding is higher compared to the studies conducted in Gonder (17.6%) and Jimma (33.4%); Ethiopia^31,48^. This could be attributed to the higher use of multiple AED therapy in our setting.

Finally, our study is not without limitations. The cross-sectional nature of the study may not provide adequate evidence of causality regarding seizure control status and its predictors. Due to self-report concerns, patients may understate socially undesirable activities like medication non-adherence and negative medication belief.

## Conclusion

Our findings revealed that more than half of the epileptic patients have uncontrolled seizure. Epileptic patients with a negative medication belief, comorbidities, low medication adherence and those who consume alcohol were more likely to have uncontrolled seizure. Therefore, particular consideration should be given to these potentially modifiable risk factors. Educational programs about the importance of their medication, adherence, and precipitating factors such as alcohol should be given. Moreover, we recommend researchers to do further longitudinal and interventional studies with more strong study design to provide adequate evidence about the cause-effect relationship between the predictor variables and seizure control.

## Data Availability

The dataset of this article is accessible on reasonable request from the corresponding author.
